# Treatment of Diphtheria—Belladonna in Spermatorrhœa

**Published:** 1878-03

**Authors:** H. L. Harrington

**Affiliations:** Warren Co., Ills.


1. *Treatment of Diphtheria.*

I have successfully treated several very severe cases of this much dreaded disease, with chlorine water, prepared as suggested by Dr. Matthew Gairdner, and used as a drink instead of water, *ad libitum*, combined with potassic chlorate, tincture of the chloride of iron, tincture of belladonnna, each ʒ iv; hydrochloric acid, strong, gtt. xx; water and simple syrup, āā ℥ i. Dose, twenty drops to one drachm, according to age, every two hours. Alternating with this a gargle was employed, composed of potassic chlorate, in saturated solution, ℥ iv, carbolic acid, gtt xx. No caustic applications were made. The false membrane disappeared much sooner (in about 36 hours), not being reproduced, and more favorable progress was made under this than any other treatment I have tried. Some of the peculiar sequelæ supervened. No deaths occurred. It will be seen that the principal agent in the above treatment is chlorine, and the younger children, who could not use the gargle, seemed to make just as rapid recovery as the others. The free administration of milk was insisted upon. No quinine or stimulants given except in one case.

2. *Belladonna in Spermatorrhœa.*

A case of the above disease of four years' standing, occurred in a farmer, aged 40, married. He was affected during cold weather, but not in summer. Voluntary erections and sexual intercourse became impossible during the continuance of the disorder, and these symptoms had persistently resisted homeopathic treatment, but yielded readily to the following: Potassic bromide, ʒ ss, fld. ext. belladonna, gtt. viij. at bedtime each night, and fld. ext. nux vomica, liq. potass. arsenit, āā gtt. v, after each meal. He was told to sleep upon a mattress instead of feathers, with light covering, to keep his mind constantly occupied, frequently changing occupation, and partake freely of nutritious unstimulating food. His condition when he applied to me was truly deplorable. Not having been able to have intercourse since last October, he suffered almost nightly from emissions, was depressed in spirits, and had loss of appetite, constipation, wandering pains, excessive palpitation of heart, in fact almost all the symptoms of an aggravated case. On the sixth night after treatment began, he having had no emissions for five nights, I advised an attempt at coition, which he successfully performed, and has repeated since. The normal habit thus being established, seems to have displaced the abnormal. He is feeling much better in every respect.

Bromide of potassium alone has not succeeded so well in my hands, perhaps because administered to single men, to whom I could not conscientiously advise sexual intercourse at stated intervals. H. L. HARRINGTON, M. D.,

Jan'y 22d, '78. *Warren Co., Ills.*

